# Drawings of Blood Cells Reveal People’s Perception of Their Blood Disorder: A Pilot Study

**DOI:** 10.1371/journal.pone.0154348

**Published:** 2016-04-28

**Authors:** Steven Ramondt, Jitske Tiemensma, Linda D. Cameron, Elizabeth Broadbent, Adrian A. Kaptein

**Affiliations:** 1 Department of Medical Psychology, Leiden University Medical Center, Leiden, The Netherlands; 2 Psychological Science, University of California Merced, Merced, California, United States of America; 3 Department of Psychological Medicine, University of Auckland, Auckland, New Zealand; University of Naples Federico II, ITALY

## Abstract

**Context:**

Sickle cell disease (SCD) and thalassemia are rare but chronic blood disorders. Recent literature showed impaired quality of life (QOL) in people with these blood disorders. Assessing one of the determinants of QOL (i.e. illness perceptions) therefore, is an important next research area.

**Objective:**

We aimed to explore illness perceptions of people with a blood disorder with drawings in addition to the Brief Illness Perception Questionnaire (Brief IPQ). Drawings are a novel method to assess illness perceptions and the free-range answers drawings offer can add additional insight into how people perceive their illness.

**Method:**

We conducted a cross-sectional study including 17 participants with a blood disorder. Participants’ illness perceptions were assessed by the Brief IPQ and drawings. Brief IPQ scores were compared with reference groups from the literature (i.e. people with asthma or lupus erythematosus).

**Results:**

Participants with SCD or thalassemia perceived their blood disorder as being more chronic and reported more severe symptoms than people with either asthma or lupus erythematosus. In the drawings of these participants with a blood disorder, a greater number of blood cells drawn was negatively correlated with perceived personal control (P<0.05), indicating that a greater quantity in the drawing is associated with more negative or distressing beliefs.

**Conclusion:**

Participants with a blood disorder perceive their disease as fairly threatening compared with people with other chronic illnesses. Drawings can add additional insight into how people perceive their illness by offering free-range answers.

## Introduction

Sickle cell disease (SCD) and thalassemia are both genetic blood disorders caused by errors in the genes for hemoglobin [1–3]. Both blood disorders have different pathophysiologies, however they do share many symptoms. Both disorders can cause stroke, fatigue, episodes of pain, and impaired quality of life (QoL) [2,4,5,6]. Two major categories of cognitive constructs can determine QoL, in addition to sociodemographic and clinical characteristics: illness perceptions and coping strategies. Both illness perceptions and coping strategies are incorporated in the Common Sense Model (CSM), which provides a framework for people’s perceptions [6]. The CSM focuses on how emotions and thoughts about an illness are formed, coping strategies are chosen, and actions are taken. People develop a cognitive representation of the disease, which shapes their coping, i.e. the way someone reacts (behaviorally, cognitively, and emotionally) to adverse events and their consequences [7]. In the CSM, people’s illness perceptions are classified within the context of several components containing specific types of information about the illness [6]: the identity/label of the illness, the expected duration (chronic/acute and cyclical), cause of illness, expected consequences, overall comprehension of the illness, emotional responses, and the ability to control the illness (both on the personal level and treatment level) [8]. Numerous empirical studies demonstrated how illness perceptions and coping strategies influence medical, psychological, and behavioral outcomes [9–11] and, in turn, QOL.

Illness perceptions are primarily measured with questionnaires, such as the Illness Perception Questionnaire (IPQ) [12], IPQ-Revised [8] and Brief IPQ [13]. A novel method to assess perceptions of the disease is with drawings. Using this method, people are asked to draw a picture of their disease, which is later assessed by identifying salient facets in those drawings [14,15]. Drawing overcome the limitations of questionnaires (which measure only predefined aspects of illness representations) by assessing the perceptions individuals have of their disease in a more direct and uncensored way [14,16–18] leading to a deeper understanding [19,20]. Earlier studies have used drawings to study perceptions of people with Cushing’s syndrome [21], myocardial infarction [22,23], heart failure [24], headache [25], melanoma [15], vestibular schwannoma [26], and systemic lupus erythematosus [18,27]. Therefore, our primary aim was to explore the illness perceptions of people with a blood disorder with drawings in addition to the Brief IPQ; furthermore, we wanted to examine interrelations between the drawings and B-IPQ scores. This pilot study will be used to gather initial data on illness perceptions of participants with a blood disorder. The results will help in designing a larger study, possibly involving an intervention to address and change illness perceptions into more adaptive/constructive ones, which may translate into improvements of quality of life or medical outcome.

A secondary aim was to compare illness perceptions of people with a blood disorder to the illness perceptions of people with other chronic illnesses, to better understand and interpret the illness perceptions of people with a blood disorder. Brief IPQ scores of people with a chronic condition (i.e. asthma or lupus) related to impaired QoL, fatigue and/or episodes of pain were chosen as the comparison groups. Brief IPQ scores for these participants were derived from the literature.

## Design and Methods

### Participants

The total number of people with SCD or thalassemia in the Netherlands is estimated to be around 800 [28]. At the 2011 annual meeting of the Dutch Sickle Cell and Thalassemia Patient Organization (OSCAR), approximately 100 people were invited to complete a short questionnaire. A short introductory talk was given at the OSCAR meeting. People were informed about the study and were asked to participate. There was sufficient time for people to ask questions before participation and everybody was aware that their data would be used for research purposes. Filling out the survey and handing it back was considered consent. All data were collected anonymously.

**Fig 1:**
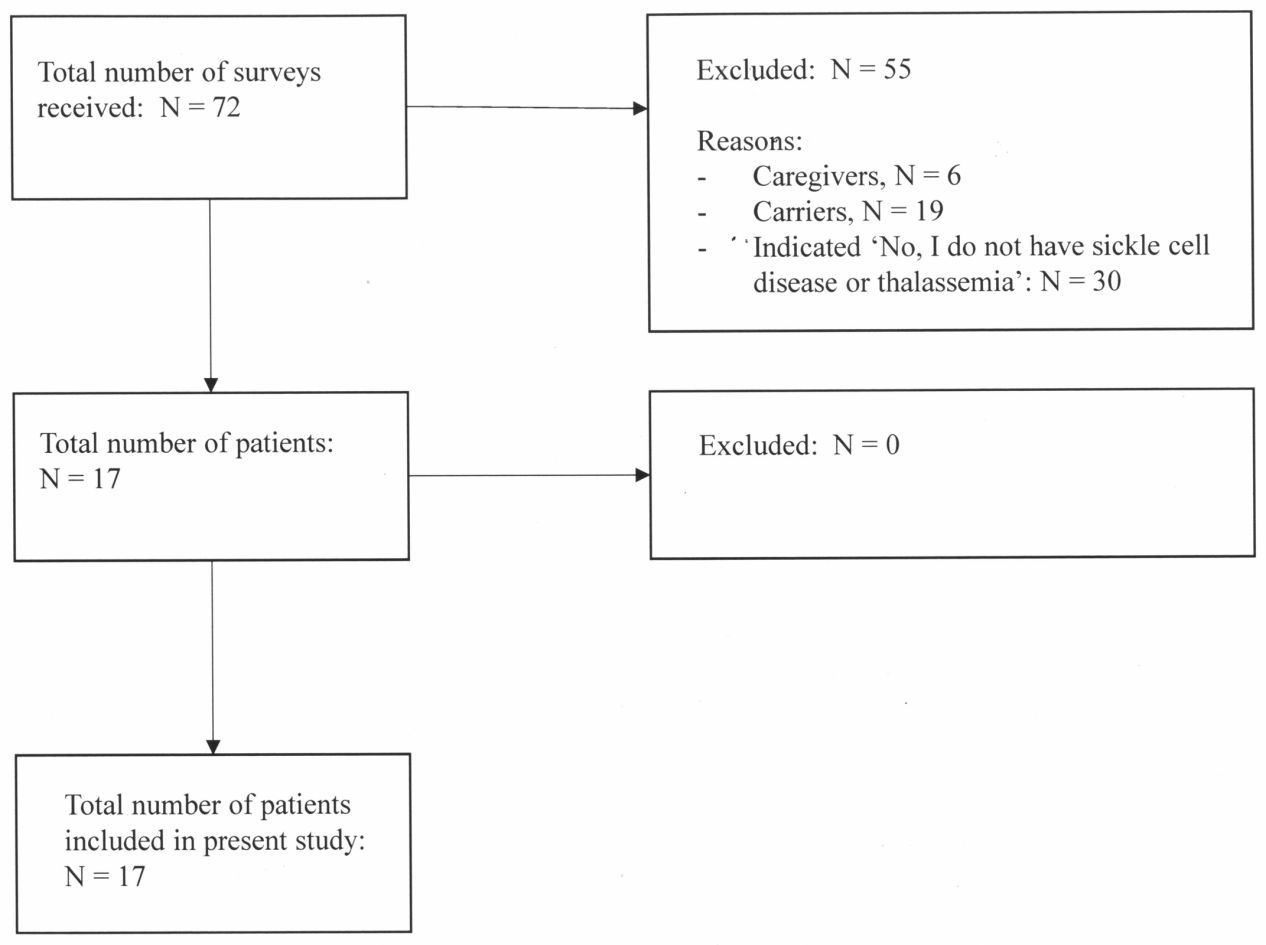
Flow chart.

The participants consisted of people with SCD, people with thalassemia, family members, partners, caregivers, health care providers and carriers of SCD or thalassemia; only people with SCD and thalassemia were included in the current study (see Fig 1). Participants were asked to list their gender, age, and type of blood disorder. In addition, the Brief Illness Perception Questionnaire (Brief IPQ) [13] and drawings were used in the current study. Seventeen participants at the meeting fulfilled the criteria (i.e. people with a blood disorder, who filled out the Brief IPQ and made drawings, in addition to providing sociodemographic characteristics). The participants were asked to refrain from disclosing any personal information (name, DOB etc.) on the survey or drawing. There were no medical doctors or hospitals involved in the design of the study or the data collection. Therefore, there are no legal obligations in the Netherlands to ask for approval of any committee.

### Questionnaire

#### Brief Illness Perception Questionnaire (Brief IPQ)

The validated Dutch language version of the Brief IPQ was used [29]. The Brief IPQ contains eight items that measure illness perceptions (on a 0–10 scale). Five of the eight items assess cognitive illness representations: Consequences (no affect at all to severely affects my life), Timeline (a very short time to forever), Personal control (absolutely no control to extreme degree of control), Treatment control (not at all to extremely helpful), and Identity (no symptoms at all to many severe symptoms). Concern (not at all concerned to extremely concerned) and Emotional response (not at all affected emotionally to extremely affected emotionally) assess the emotional representations, and Understanding (don’t understand at all to understand very clearly) assesses illness comprehensibility, i.e. the way in which people make sense of their illness. To measure Cause, participants responded to an open-ended question about what they believe are the three most important causes for their illness [13]. Means and standard deviations were calculated for the Brief IPQ items. Answers to the open-ended question were grouped in three categories, i.e., inheritance, perceived symptoms and other causes.

#### Drawings

Instructions about the drawing were similar to Broadbent et al. (23). Participants were given a one page questionnaire on a A3-piece of paper containing the B-IPQ items and two standard 10 x 10 centimeter boxes to draw in. The participants were than given the following instructions: “Please draw a picture of a drawing of the blood from someone without SCD/thalassemia, and another picture of the blood from someone with SCD/thalassemia. This task is not about artistic ability- simple sketches are fine. We are interested in your own ideas about the blood of somebody with SCD/thalassemia”. Participants were asked to draw with a pencil or pen.

### Reference groups

The comparison groups included people with asthma (N = 309) [13] and people with lupus (N = 106) [30]. The group of participants with asthma were recruited from general practitioner clinics in the United Kingdom. Participants had a mean age of 39.8 (SD = 10.1); 58.9% were female. The mean length of illness was 22.3 years (SD = 13.4 years). Their Brief IPQ scores were derived from Broadbent et al. [13]. The participants with lupus erythematosus were recruited from the rheumatology clinic at an outpatient clinic of Auckland City Hospital, and from two lupus patients’ association in New Zealand. All participants were receiving treatment with corticosteroids and/or another immunosuppressive agent. One hundred and forty-one people were approached, and 106 people were willing to participate. Participants had a mean age of 43.3 (SD = 15) with 94% being female. The mean disease duration was 10.2 years (SD = 9.1 years). Their Brief IPQ scores were derived from Daleboudt et al. [30].

### Data analysis

The drawings were scanned and independent observers scored the characteristics of the drawing. The procedures to identify categories were identical to previous drawing research [23]. Prominent facets of the drawings were identified. These facets were used to develop a coding framework. Two coders then independently coded the drawings, i.e. how many healthy blood cells and irregular blood cells were drawn, emotion in the drawing (present or absent), and present or absent visible pain in the drawing (since pain is a common symptom of SCD). Furthermore, present or absent written explanation underneath the drawing was scored by both observers. Kappa scores were calculated for each scored characteristic. Consensus was reached between the raters when the Kappa was <0.7. This was done through reanalyzing the drawings by the two raters.

Data were analyzed using PASW Statistics version 17.0.2 (SPSS Inc., Chicago, IL). The dataset can be found in S1 Dataset.

Data are shown as mean (SD) unless mentioned otherwise. The primary analysis comprised the comparison of results in people with SCD/thalassemia versus various reference groups. The means were compared between groups using a Student’s t-test. The Kolmogorov-Smirnov test was used to check the normality of the data, while Levene’s test was used to check the equality of variance between groups. The variables Consequences, Timeline, and Understanding were not normally distributed in our sample. A linear regression analysis (method: enter) was performed to explore the impact of gender and age on illness perceptions. The first and second drawing were compared using paired t-tests for the continuous variables (number of healthy cells and number of irregular cells), and χ^2^ for the categorical variables. To investigate the association between characteristics of the drawings and illness perceptions, Spearman’s rho correlation coefficient was used. χ^2^ was used in event of categorical data. All tests were two-tailed and the level of significance for this analysis was set at p≤0.05.

## Results

The sample consisted of five males and twelve females with a mean age of 33.6 (SD = 3.6) yr. Ten participants were diagnosed with sickle cell disease, and seven participants with thalassemia. People with different types of blood disorders (i.e. SCD and thalassemia) did not significantly differ from each other on any of the Brief IPQ items. The mean scores on the eight Brief IPQ dimensions for these participants with a blood disorder are listed in Table 1 and can be found in the S1 Dataset. When using a linear regression model, gender (coded as male = 0, female = 1) was associated with identity (β = 0.464, p = 0.002), concern (β = 0.446, p = 0.011), and emotional response (β = 0.469, p = 0.006). This indicates that females score higher on these subscales of the Brief IPQ, and thus report a stronger impact of the blood disorder on their lives, reported stronger concerns, and stronger emotional responses. Furthermore, age was associated with identity (β **=** -0.439, p = 0.004), indicating that older participants scored lower on identity and perceived less of an impact of the disease on their lives.

**Table 1:** Scores (means, standard deviation) on Brief IPQ of the participants with SCD and thalassemia, compared with two reference groups.

Brief IPQ	Blood disorder, n = 17	Asthma^1^, n = 309	Lupus^2^, n = 106
Consequences^3^	**7.6 (1.9)**	3.5 (2.3)***	5.45 (2.7)***
Timeline	**9.9 (0.3)**	8.8 (2.2)***	8.4 (2.5)***
Personal control	**6.1 (2.7)**	6.7 (2.4)	4.9 (3.0)
Treatment control	**6.9 (2.3)**	7.9 (2.0)*	2.7 (2.2)***
Identity^3^	**7.2 (2.5)**	4.5 (2.3)***	6.1 (2.6)
Concern^3^	**7.2 (2.4)**	4.6 (2.8)***	6.9 (2.8)
Understanding	**6.8 (3.6)**	6.5 (2.6)	3.3 (2.5)**
Emotional response^3^	**6.7 (2.8)**	3.3 (2.9)***	5.5 (3.0)

* p<0.05 compared to participants with SCD

** p<0.01 compared to participants with SCD

*** p<0.001 compared toparticipants with SCD.

^1^Values from Broadbent et al. [13].

^2^Values from Daleboudt et al. [30].

^3^Higher scores indicate more negative perceptions.

The participants’ perceptions about the most important cause for their disease were grouped in three categories. ‘Inheritance’ was mentioned by 41% (N = 7) of the respondents, people described ‘perceived symptoms’ (for example, stress and pain) in 24% (N = 4) of the cases, and ‘other causes’ was mentioned by 18% (N = 3). Three participants did not list a possible cause for their disease.

### Illness perceptions of people with a blood disorder compared with reference groups

Compared with the people with asthma, the illness perceptions of participants with a blood disorder were more negative on six out of eight dimensions. They scored more negative on Consequences (p<0.001), Timeline (p<0.001), Treatment Control (p<0.05), Identity (p<0.001), Concern (p<0.001) and Emotional response (p<0.001). In comparison with people with lupus erythematosus, the participants with a blood disorder scored more negative on Consequences (p<0.001) and Timeline (p<0.001), but people with a blood disorder scored more positive on Treatment Control (p<0.001) and Understanding ((p<0.01). The results are listed in Table 1.

### Drawings

**Fig 2:**
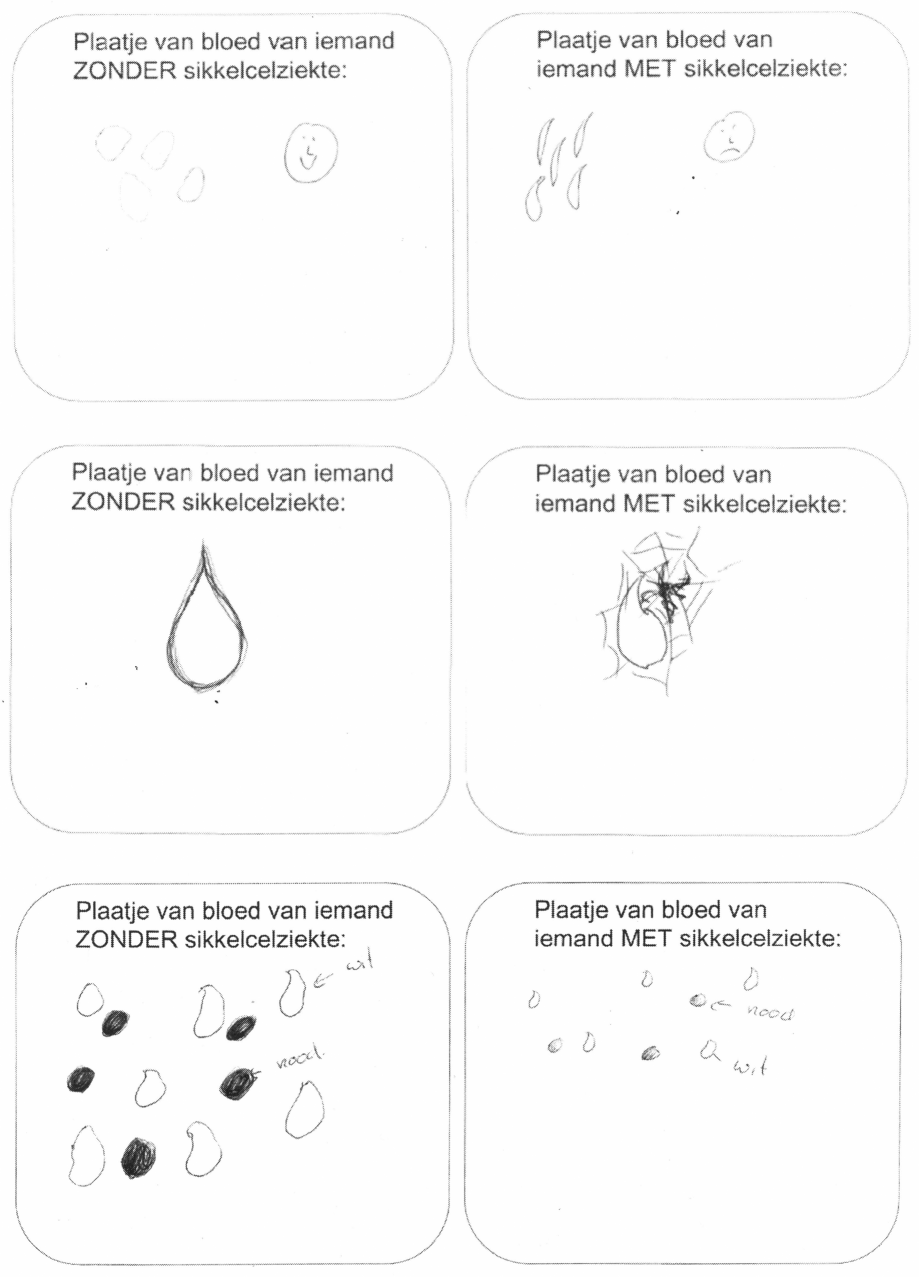
Examples of drawings by participants with a blood disorder. Translation drawing 1: Picture of blood of someone without SCD. Translation writing in drawing 1c: Dutch: wit; English: white, Dutch: rood; English: red. Translation drawing 2: Picture of blood of someone with SCD. Translation writing in drawing 2c: Dutch: wit; English: white, Dutch: rood; English: red. Drawings participant A: female, 39 years. Drawings participant B: female, 26 years. Drawings participant C: female, 25 years.

Some examples of drawings are shown in Fig 2. The first drawing represents the perception of participants when asked to make a drawing of the blood from someone *without* SCD/thalassemia. The second drawing shows the perception of participants when asked to make a drawing of the blood from someone *with* SCD/thalassemia. Written explanations under or in both drawings included for example the word ‘Thalassemia’ or arrows with the words ‘white cells’ or ‘red cells’ to indicate the kind of cells drawn. Other written explanations included emotions (e.g. ‘sad’ or ‘smile’), or properties of the different cells (e.g. ‘normal size’ and ‘nice and round’, or ‘broken cells’ and ‘smaller cells’).

When asked to make a drawing of the blood from someone *without* SCD/thalassemia almost everyone (94%, n = 16) drew blood cells. All of the cells that participants drew were healthy cells, i.e., with a round, regular shape. Almost 18% (n = 3) of the participants showed a positive emotion in their drawing of healthy cells; e.g., blood cells with smiling faces. Not one participant drew pain in any shape or form. Six of the participants (35%) wrote an explanation underneath their drawing. In the second drawing, displaying blood from someone *with* SCD/thalassemia, the same number of participants drew blood cells (94%, n = 16). Six percent (n = 1) of the participants drew only healthy cells, 65% (n = 11) drew only irregular cells, and almost 24% (n = 4) of the participants drew both healthy as well as irregular cells. Nearly 24% (n = 4) drew some sort of emotion, all of it negative, such as sad faces. Nearly 24% (n = 4) of the participants explained their drawing in writing. Again, nobody drew any form of pain.

When the drawings were compared, the drawing of blood without SCD showed more healthy cells (6.6 (SD = 9.0) versus 2.1 (SD = 5.0)), but fewer irregular cells (0.0 (SD = 0.0) versus 3.9 (SD = 3.6)) than the drawing of blood with SCD (both p<0.001) although there were no differences in the total number of cells drawn (6.6 (SD = 9.0) versus 6.0 (SD = 5.9)). Furthermore, there was more negative emotion visible in the drawing of blood with SCD (χ^2^ = 24.7, p<0.001) and less written explanation (χ^2^ = 16.3, p<0.001) compared with the drawing of blood without SCD.

### Association between drawings and illness perceptions

Perceived personal control was negatively correlated with the total number of blood cells in the first drawing of blood without SCD (r = -.58, p = 0.014), the number of healthy blood cells in the drawing of blood without SCD (r = -.58, p = 0.014), and the total number of blood cells in the drawing of blood with SCD ((r = -.49, p = 0.047). These results show a consistent pattern in which drawing more blood cells is associated with lower beliefs of personal control over the blood disorder. There were no significant correlations between visible emotion or written explanation and any of the Brief IPQ dimensions.

## Discussion

This pilot study explored illness perceptions of participants with a blood disorder. In addition, we investigated the differences in illness perceptions between these participants with blood disorders and two reference groups.

In general, participants with a blood disorder had more negative illness perceptions compared with both reference groups (i.e. asthma, lupus erythematosus). Participants perceived their blood disorder as being more chronic and as having more severe symptoms than people with either asthma or lupus erythematosus. Furthermore, compared with people with asthma, participants with blood disorders perceived that the illness had a greater effect on their lives, they were more concerned about the illness and were more emotionally affected by it. On the other hand, they perceived greater treatment control over their illness and understood their illness better than people with lupus erythematosus. These perceptions may reflect the permanence and severity of these chronic blood disorders. People with SCD have a low life expectancy; the average life expectancy of males is estimated to be 42 years and 48 years for females [31]. In addition, people with thalassemia have to undergo intense treatment (i.e., blood transfusions). When exploring the possible cause of their blood disorder (i.e., Causes in Brief IPQ), almost half of the participants reported 'inheritance' as most important cause. This shows that participants seem to understand what caused their blood disorder, which is in line with their higher scores on the understanding dimension of the B-IPQ compared to the reference groups.

Participants’ drawings of blood cells of someone without a blood disease and of someone with a blood disease revealed how participants visualized the illness in a pictorial manner. In drawings of participants with SCD and participants with thalassemia, a greater number of blood cells drawn was associated with poorer perceived personal control. These findings are comparable to earlier studies [25,27], and suggest that a greater magnitude (quantity or size) of an affected body part is associated with more negative or distressing beliefs. One might expect that drawing more blood cells would be associated with higher perceived control over the condition, but this depends on the assumption that people understand that the disease destroys blood cells. The findings of this study may illustrate that people do not understand this, and instead they think the more blood cells the worse the condition. An alternative explanation is that people who experience worse illness severity/symptoms perceive lower control and experience their disease as more prominent in their lives and therefore draw more blood cells. This explanation is supported by previous research that has shown people tend to draw objects larger when the objects are more salient to them [32]. We did not find any other association between the characteristics of the drawings and the Brief IPQ. This is in contrast to earlier studies, which found more associations between drawings and illness perceptions [16,17,19, 20]. One reason for this discrepancy may be that the participants were asked to draw the blood cells of “someone” with the blood disorder, whereas most other drawings studies have asked participants to draw themselves. This means that the drawings in this study may not reflect the participants’ perceptions of how the condition affects them personally but reflect their understanding of the condition in the average person. A similar result to the current study was found by Tiemensma et al. [21], who reported that the drawings of people after long-term remission of Cushing’s syndrome showed almost no associations with illness perceptions. It is possible that drawings reflect a new dimension of the psychological status that is not assessed in standard questionnaires [21].

In the current study, a third of the participants drew negative emotions in the drawings of the disorder, which is in line with their ratings of being highly emotionally affected by the disorder. No participants drew any form of pain, which may be because the instructions were quite specific in asking participants to draw blood. If the participants were asked to draw how their illness affected them, they may have been more likely to draw pain or lifestyle restrictions [33]; future work should aim to investigate this.

The current study has some limitations, making our study a pilot exploration of a relatively unexplored subject. The small sample size–due to the low prevalence of SCD and thalassemia- limits the power of the study and limits the generalizability of the results. The low power in this study might be responsible for not detecting more associations between drawing characteristics and the Brief IPQ. In addition, it would have been useful to collect more demographic information. Future studies should take these limitations into account when designing a similar study. In addition, the reference groups were not matched with our sample.

In conclusion, participants with SCD or thalassemia perceive their disease as quite threatening compared with people with other chronic illnesses, especially asthma. Drawings can add additional insight into how people perceive their illness by offering free-range answers. The Brief IPQ and drawings are quick and inexpensive to administer and easy to collect. They can be used as a starting point in providing information, which is helpful in the context of encouraging self-management and facilitating empowerment. They can also be used to broaden the subject of emotional responses to the illness, which often goes ignored in the clinical encounter. This gives people the opportunity to discuss his/her feelings and perceptions with a physician and any misconceptions can be addressed, which in turn may be instrumental in improving QOL.

## Supporting Information

S1 Dataset Dataset containing data on perceptions and drawings of blood disorders (SAV)

## References

[1] van den Tweel XW, Hatzmann J, Ensink E, van der Lee JH, Peters M, Fijnvandraat K, et al Quality of life of female caregivers of children with sickle cell disease: a survey. Haematologica. 2008;93: 588–93. 10.3324/haematol.11610 18322259

[2] Ataga KI, Cappellini MD, Rachmilewitz EA. Beta-thalassaemia and sickle cell anaemia as paradigms of hypercoagulability. Br J Haematol. 2007;139: 3–13. 17854302 10.1111/j.1365-2141.2007.06740.x

[3] Weatherall DJ. The thalassaemias. BMJ. 1997;314: 1675–8. 9193293 10.1136/bmj.314.7095.1675 PMC2126866

[4] Steinberg MH. Management of sickle cell disease. N Engl J Med. 1999;340: 1021–30. 10099145 10.1056/NEJM199904013401307

[5] Gabutti V, Borgna-Pignatti C. Clinical manifestations and therapy of transfusional haemosiderosis. Baillieres Clin Haematol. 1994;7: 919–40. 7881160 10.1016/s0950-3536(05)80131-3

[6] Leventhal H, Brissette I, Leventhal EA. The common-sense model of self-regulation of health and illness In: Cameron LD, Leventhal H, editors. The self-regulation of health and illness behaviour. London: Routledge; 2003 pp. 42–65.

[7] Schreurs PGJ, van de Willige G, Brosschot JF, Tellegen B, Graus GHM.). De Utrechtse Coping Lijst: UCL [Manual]. Lisse, The Netherlands; 1993.

[8] Moss-Morris R, Weinman J, Petrie K, Horne R, Cameron L, Buick D. The Revised Illness Perception Questionnaire (IPQ-R). Psychol Health. Routledge; 2002;17: 1–16.

[9] Scharloo M, Kaptein AA, Weinman J, Hazes JM, Willems LN, Bergman W, et al Illness perceptions, coping and functioning in patients with rheumatoid arthritis, chronic obstructive pulmonary disease and psoriasis. J Psychosom Res. 1998;44: 573–85. 9623878 10.1016/s0022-3999(97)00254-7

[10] Petrie KJ, Jago LA, Devcich DA. The role of illness perceptions in patients with medical conditions. Curr Opin Psychiatry. 2007;20: 163–7. 17278916 10.1097/YCO.0b013e328014a871

[11] Vogel JJ, Godefroy WP, van der Mey AGL, le Cessie S, Kaptein AA. Illness perceptions, coping, and quality of life in vestibular schwannoma patients at diagnosis. Otol Neurotol. 2008;29: 839–45. 10.1097/MAO.0b013e3181820246 18636026

[12] Weinman J, Petrie KJ, Moss-Morris R, Horne R. The Illness Perception Questionnaire: A new method for assessing the cognitive representation of illness. Psychol Health. Routledge; 1996;11: 431–445.

[13] Broadbent E, Petrie KJ, Main J, Weinman J. The brief illness perception questionnaire. J Psychosom Res. 2006;60: 631–7. 16731240 10.1016/j.jpsychores.2005.10.020

[14] Broadbent E, Ellis CJ, Gamble G, Petrie KJ. Changes in patient drawings of the heart identify slow recovery after myocardial infarction. Psychosom Med. 2006;68: 910–3. 10.1097/01.psy.0000242121.02571.10 17079705

[15] Scott SE, Birt L, Cavers D, Shah N, Campbell C, Walter FM. Patient drawings of their melanoma: a novel approach to understanding symptom perception and appraisal prior to health care. Psychol Health. 2015;30: 1035–48. 10.1080/08870446.2015.1016943 25674833

[16] Salmon PL. Viewing the client’s world through drawings. J Holist Nurs. 1993;11: 21–41. 8450185 10.1177/089801019301100104

[17] Guillemin M. Understanding illness: using drawings as a research method. Qual Health Res. 2004;14: 272–89. 14768462 10.1177/1049732303260445

[18] Nowicka-Sauer K. Patients’ perspective: lupus in patients' drawings. Assessing drawing as a diagnostic and therapeutic method. Clin Rheumatol. 2007;26: 1523–5. 17447104 10.1007/s10067-007-0619-9

[19] Ångström-Brännström C, Norberg A. Children undergoing cancer treatment describe their experiences of comfort in interviews and drawings. J Pediatr Oncol Nurs. 2014;31: 135–46. 10.1177/1043454214521693 24651546

[20] Woodgate RL, West CH, Tailor K. Existential anxiety and growth: an exploration of computerized drawings and perspectives of children and adolescents with cancer. Cancer Nurs. 2014;37: 146–59. 10.1097/NCC.0b013e31829ded29 24145247

[21] Tiemensma J, Daskalakis NP, van der Veen EM, Ramondt S, Richardson SK, Broadbent E, et al Drawings reflect a new dimension of the psychological impact of long-term remission of Cushing’s syndrome. J Clin Endocrinol Metab. 2012;97: 3123–31. 10.1210/jc.2012-1235 22723334

[22] Broadbent E, Ellis CJ, Gamble G, Petrie KJ. Changes in patient drawings of the heart identify slow recovery after myocardial infarction. Psychosom Med. 2006;68: 910–3. 17079705 10.1097/01.psy.0000242121.02571.10

[23] Broadbent E, Petrie KJ, Ellis CJ, Ying J, Gamble G. A picture of health—myocardial infarction patients’ drawings of their hearts and subsequent disability: a longitudinal study. J Psychosom Res. 2004;57: 583–7. 15596165 10.1016/j.jpsychores.2004.03.014

[24] Reynolds L, Broadbent E, Ellis CJ, Gamble G, Petrie KJ. Patients’ drawings illustrate psychological and functional status in heart failure. J Psychosom Res. 2007;63: 525–32. 17980226 10.1016/j.jpsychores.2007.03.007

[25] Broadbent E, Niederhoffer K, Hague T, Corter A, Reynolds L. Headache sufferers’ drawings reflect distress, disability and illness perceptions. J Psychosom Res. 2009;66: 465–70. 10.1016/j.jpsychores.2008.09.006 19379963

[26] van Leeuwen BM, Herruer JM, Putter H, van der Mey AGL, Kaptein AA. The art of perception: Patients drawing their vestibular schwannoma. Laryngoscope. 2015;125: 2660–2667. 10.1002/lary.25386 26059643

[27] Daleboudt GMN, Broadbent E, Berger SP, Kaptein AA. Illness perceptions in patients with systemic lupus erythematosus and proliferative lupus nephritis. Lupus. 2011;20: 290–8. 10.1177/0961203310385552 21362752

[28] Giordano PC, Bouva MJ, Harteveld CL. A confidential inquiry estimating the number of patients affected with sickle cell disease and thalassemia major confirms the need for a prevention strategy in the Netherlands. Hemoglobin. 2004;28: 287–96. 15658185 10.1081/hem-200037735

[29] de Raaij EJ, Schröder C, Maissan FJ, Pool JJ, Wittink H. Cross-cultural adaptation and measurement properties of the Brief Illness Perception Questionnaire-Dutch Language Version. Man Ther. 2012;17: 330–5. 10.1016/j.math.2012.03.001 22483222

[30] Daleboudt GMN, Broadbent E, McQueen F, Kaptein AA. The impact of illness perceptions on sexual functioning in patients with systemic lupus erythematosus. J Psychosom Res. 2013;74: 260–4. 10.1016/j.jpsychores.2012.11.004 23438719

[31] Platt OS, Brambilla DJ, Rosse WF, Milner PF, Castro O, Steinberg MH, et al Mortality in sickle cell disease. Life expectancy and risk factors for early death. N Engl J Med. 1994;330: 1639–44. 7993409 10.1056/NEJM199406093302303

[32] Craddick RA. Height of Christmas tree drawings as a function of time. Percept Mot Skills. 1963;17: 335–9. 14057238 10.2466/pms.1963.17.2.335

[33] Chong J, Mackey AH, Stott NS, Broadbent E. Walking drawings and walking ability in children with cerebral palsy. Heal Psychol. 2013;32: 710–3. 10.1037/a0027353 22369490

